# Cardiac MRI structural and functional predictors of left ventricular ejection fraction recovery following PVC catheter ablation

**DOI:** 10.1038/s41598-021-87754-2

**Published:** 2021-04-15

**Authors:** Jessica Mao, Eric Xie, Ela Chamera, Joao A. C. Lima, Jonathan Chrispin

**Affiliations:** Division of Cardiology, The Johns Hopkins Hospital, Baltimore, MD USA

**Keywords:** Arrhythmias, Cardiomyopathies

## Abstract

Frequent premature ventricular contractions (PVCs) can induce cardiomyopathy (PVC CM). We sought to use cardiac magnetic resonance imaging (CMR) to quantify changes in cardiac structure and function of cardiomyopathy patients following catheter ablation for PVCs. Patients undergoing PVC ablation at the Johns Hopkins Hospital with pre-procedural CMR from 2010 to 2018 were included in this study. CMR Images were analyzed to collect information on cardiac structure and function as well as to quantify scar. Of the total 51 included patients, PVC CM (LVEF < 45%) was observed in 51% (n = 29). Of these, 19 had post-ablation ejection fractions quantified, with 78.9% (n = 15) recovering function. Global longitudinal strain was significantly correlated with LVEF (OR 1.831, *p* < 0.01) but did not predict recovery of function. RV origin of PVCs was more common in the preserved LVEF group but was also significantly correlated with persistently reduced EF post-ablation in the PVC CM group. Scar burden was not correlated with either cardiac function or post-ablation recovery of function. In this cohort, there were no significant CMR findings to predict subsequent recovery of EF after ablation among those with PVC CM. PVC origin in the RV was associated with persistently reduced LVEF after ablation.

## Introduction

Premature ventricular contractions (PVCs), while historically thought benign, are now well recognized to carry risk of inducing cardiomyopathy. There have been significant efforts to isolate predictors of which patients will go on to develop PVC-CM, some isolating PVC burden^1–5^, PVC morphology^6–9^, or gender as risk factors. PVC CM is often a diagnosis of exclusion, and reversibility with resolution of PVCs, often by radiofrequency ablation, separates it from other underlying cardiomyopathy. Few prior studies used cardiac magnetic resonance imaging (CMR) assess for presence of SHD with relation to improvement of cardiac function after PVC ablation.

We sought to use CMR to quantify left ventricular scar and function in patients undergoing catheter ablation for PVCs, and to identify imaging parameters associated with recovery of ejection fraction after successful ablation.

## Methods

### Patient selection

Patients who had undergone PVC ablation at the Johns Hopkins Hospital between 2010 and 2018 were selected. These patients had been shown to have PVC either by Holter monitoring or via inpatient telemetry. Rationale for undergoing PVC ablation included symptomatic palpitations, pre-syncope or syncope, or cardiomyopathy of unknown etiology. Of patients undergoing PVC ablation, those with CMR completed within 6 months pre-procedure were selected.

Patients with previously known coronary artery disease (CAD) or valvular structural heart disease (SHD) were excluded. Ischemic evaluation was performed prior to the CMR. When CMR findings suggested alternative diagnosis, such as arrhythmogenic right ventricular cardiomyopathy (ARVC) or cardiac sarcoidosis, these patients were also excluded. However, patients discovered to have scar appearing ischemic in etiology during image analysis were still included.

### Definitions

Cardiomyopathy was defined as ejection fraction (EF) < 50%, which was taken from the most recent TTE prior to ablation. Recovery of EF was defined as improvement > 10% after ablation.

### Clinical data

This was a retrospective cohort study. Retrospective review of patient data was approved by the Johns Hopkins University Institutional Review Board. All procedures were carried out in accordance with the relevant guidelines. Written informed consent was obtained from all participants to have clinical and personal data available for in research purposes.

The electronic medical record was used to collect participant demographic data, presenting symptoms, PVC burden (if Holter had been performed prior to procedure), anti-arrhythmic medications prior to procedure, most recent TTE prior to and after ablation, known presence of structural heart disease, and known history of coronary artery disease.

### Electrophysiology study and radiofrequency ablation

Electrophysiology study (EPS) information was extracted from procedural notes in the medical record, including number of PVCs and their morphologies, number of ablated sites and locations, as well as procedural success. Acute procedural success was defined as the post-procedure absence of PVCs after a 30 min observation period.

### Cardiac MRI

Not all patients had CMR images obtained at the Johns Hopkins Hospital, and many images were acquired at outside facilities. Thus, CMR images were obtained from all major vendors (Siemens, Philips, and General Electric) at both 1.5 and 3 T. A complete sequence included cine sequences in both the short axis, long-axis, 3 chamber views with intervals of roughly 1 cm across the left ventricle, as well as delayed enhancement imaging. CMR images were analyzed using QMass 7.5 (Medis, Leiden, The Netherlands). Information on cardiac structure, including left ventricular end diastolic volume (LVEDV), cardiac mass, scar burden, as well as on cardiac function, including cardiac output/index, global longitudinal strain and left ventricular ejection fraction were collected. Delayed enhacement CMR (DE-CMR) was used to identify scar. Scar was identified as myocardium with abnormal enhacement with signal intensity at least 2 standard deviations above identified normal intensity tissue. Scar mass and volume were then indexed as percentage of the LV myocardium.

Quantification of LV strain was accomplished using multimodality tissue tracking software (MTT, Toshiba Medical Systems). Global longitudinal strain was assessed using cine-CMR in the 4-chamber view. Endocardial and epicardial contours were manually drawn at end-systole and automatically propagated through the cardiac cycle, with manual correction as needed. Strain and strain rate were then calculated within the software by tracking the position of index pixels.

### Statistical analysis

Statistics were completed using IBM SPSS Statistics v25 (IBM, New York, USA). Group differences were compared with Fisher’s exact test for categorical variables and t-test for continuous variables. Presence of cardiomyopathy was defined as a binary variable, and binomial logistic regression was performed to assess whether age, gender, medication use, PVC burden, number of PVC morphologies, whether ablation was able to be successfully performed, ablation site, presence of scar, scar burden, maximum longitudinal strain, and maximum strain rate (SR) were each associated. Next, ejection fraction was addressed as a continuous variable and ordinary least squares linear regressions were performed to assess the relation between the same variables and EF.

Of the patients with cardiomyopathy, those with follow-up assessment of cardiac function were assessed. Using recovery of ejection fraction as a binary variable, binomial logistic regressions were performed to assess whether the same variables were associated with recovery.

For evaluation of longitudinal strain, Pearson correlation was performed. A *p* value < 0.05 was considered statistically significant for all statistics.

## Results

### Baseline characteristics

**Figure 1:**
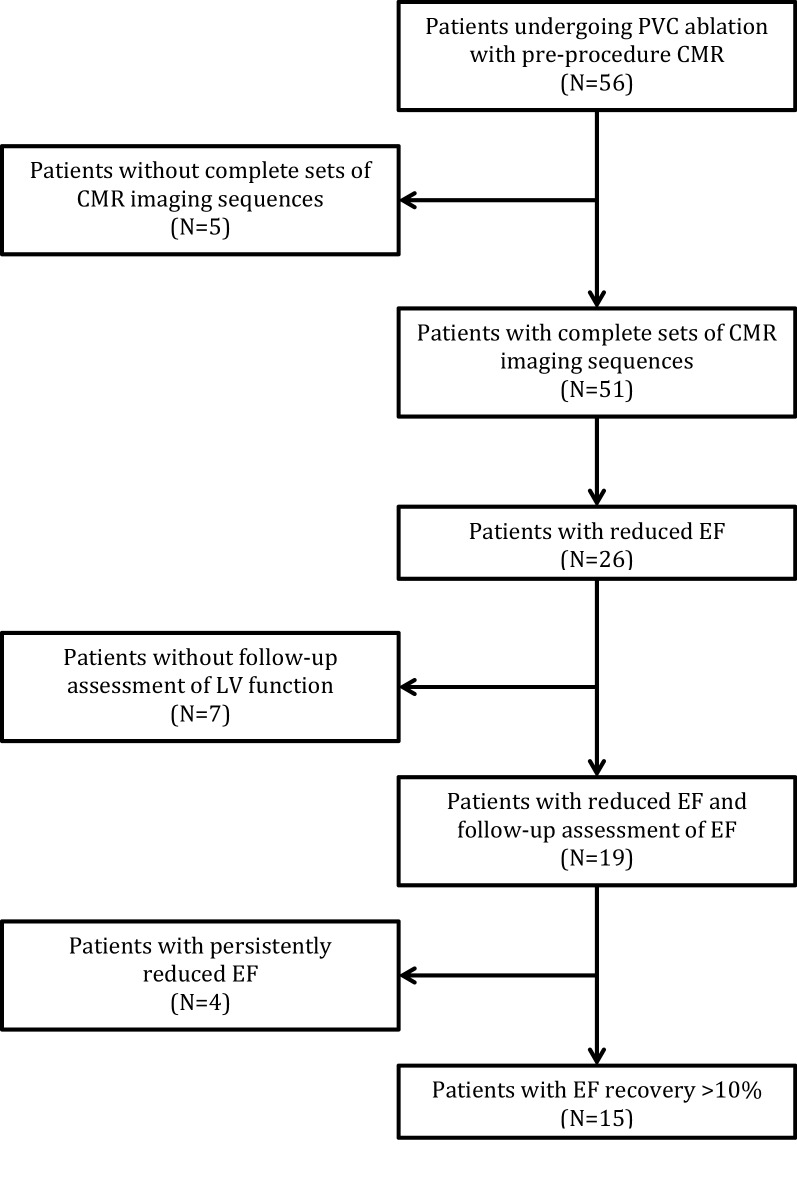
Flowchart of inclusionary criteria.

Between 2010 and 2018, a total of 56 patients undergoing PVC ablation also had CMR images available prior to ablation. CMR images were obtained on average 1 week prior to the ablation procedure. Of these, 51 patients had complete sets of images for analysis. Cardiomyopathy was observed in 26 patients (51.0%). Of these 26 patients, 19 had follow-up assessment of cardiac function on file. Post ablation follow-up echocardiograms were obtained on average 18 months post-ablation. Fifteen (78.9%) of these patients had recovery of EF after ablation (Fig. 1).

In comparing the cardiomyopathy patients to those with preserved EF, there were no significant differences in demographics or medical history (Table 1).

**Table 1:** Baseline characteristics of study patients.

	Total (n = 51)	Reduced EF (n = 26)	Normal EF (n = 25)	*p*
Age	52.12 ± 16.41	51.46 ± 16.99	52.8 ± 16.10	0.57
Male	24 (47.06%)	14 (53.85%)	10 (40%)	0.40
Pre-ablation EF (%)	49.98 ± 12.27	40.35 ± 8.57	60 ± 5.66 (24%)	**< 0.001**
**Medication**				
BB	30 (58.82%)	18 (69.23%)	12 (48%)	0.16
CCB	6 (11.76%)	1 (3.85%)	5 (20%)	0.09
Class IC	4 (7.84%)	3 (11.54%)	2 (8%)	0.61
Class III	3 (5.88%)	1 (3.85%)	2 (8%)	0.67
PVC burden (%)	22 ± 10.4%	23 ± 10.91%	21 ± 9.92%	0.49
Number of PVC morphologies	1.51 ± 1.19	1.54 ± 1.42	1.48 ± 0.92	0.13
Unsuccessful ablation	8 (15.69%)	5 (19.23%)	3 (12%)	0.70
**Ablation site**				
LV	21 (41.18%	14 (53.85%)	7 (28%)	0.09
RV	23 (45.10%)	9 (34.62%)	14 (56%)	**0.03**
Both	4 (7.84%)	1 (3.85%)	3 (12%)	0.13
LVEDV (mL)	158.66 ± 63.19	175.43 ± 77.22	140.50 ± 36.93	**0.04**
LVEDVi	79.80 ± 27.91	85.35 ± 34.02	73.79 ± 18.14	0.13
Scar	10 (19.6%)	5 (19.23%)	5 (20%)	0.94
Scar burden (%)	11.7 ± 7.18	15.33 ± 7.23	9.0 ± 7.09	0.56
Global longitudinal strain (%)	− 18.32 ± 4.91	− 16.19 ± 4.50	20.62 ± 4.34	**< 0.001**
Max strain rate	1.85 ± 0.82	1.85 ± 0.89	1.84 ± 0.75	0.21

bolded values represent statistically significance with *p*< 0.05.

### CMR findings

#### Scar presence

Of the total cohort, 10 patients (19.6%) had delayed enhancement on CMR. There was an equal number of patients with scar present in both the reduced EF and the preserved EF group (5 in each). The average scar burden was 15.3 ± 7.23% of the reduced EF group and 9.0 ± 7.09% in the preserved EF group (*p* = 0.3). There as no significant correlation between either the presence of scar or scar burden with EF (Table 2).

**Table 2:** Characteristics of cardiomyopathy patients with results of linear regression.

	Recovered EF (n = 15)	Persistently reduced EF (n = 4)	*p*	OR (95% CI)	*p*
Age	57.67 ± 16.00	41.5 ± 15.59	0.13	1.05 (0.98–1.12)	0.19
Male	9 (60%)	1 (25%)	0.27	7.20 (0.11–1.25)	0.11
Pre-ablation EF (%)	38.73 ± 7.70	39.5 ± 14.27	0.91	0.99 (0.83–1.11)	0.83
**Medication**					
BB	12 (80%	1 (25%)	0.39	9.00 (0.87–92.76)	0.06
CCB	1 (6.67%)	0 (0%)	0.33	–	–
Class IC	1 (6.67%)	0 (0%)	0.33	–	–
Class III	1 (6.67%)	1 (25%)	0.52	–	–
PVC burden (%)	20.91 ± 9.80	27.00 ± 10.58	0.43	0.89 (0.76–1.04)	0.13
Number of PVC morphologies	1.27 ± 0.46	1.75 ± 0.96	0.39	0.42 (0.08–2.29)	0.32
Unsuccessful ablation	5 (33%)	0 (0%)	0.02	–	–
**Ablation site**					
LV	10 (66.67%)	1 (25%)	0.20	2.70 (0.33–21.98)	0.35
RV	2 (13.33%)	3 (75%)	0.08	0.11 (0.01–1.15)	0.06
Both	1 (6.67%)	0 (0%)	0.33	–	–
LVEDVi	81.28 ± 20.60	73.33 ± 24.32	0.50	1.00 (0.99–1.01)	0.61
Scar	2 (13.33%)	1 (25%)	0.68	0.67 (0.05–9.47)	0.76
Scar burden (%)	13 ± 8.49	5	0.80	1.05 (0.79–1.40)	0.72
Global longitudinal strain (%)	− 16.54 ± 4.45	− 15.15 ± 5.53	0.67	1.03 (0.82–1.29)	0.82
Max strain rate	1.91 ± 9.36	1.53 ± 0.79	0.44	1.28 (0.39–4.19)	0.68

Within the cardiomyopathy group, presence of scar was not significantly correlated with recovery of EF (Table 3).

**Table 3:** Correlation of patient data with PVC cardiomyopathy and on ejection fraction.

	Ejection fraction (continuous)		PVC CM (binary)	
	OR (95% CI)	*p*	OR (95% CI)	*p*
Age	0.91 (0.74–1.13)	0.40	1.75 (0.58–5.32)	0.324
Male	0.01 (8.36 × 10^–6^–7.44)	0.16	0.99 (0.96–1.03)	0.77
**Medication**				
BB	0.02 (2.15 × 10^–6^–1.97)	0.08	2.44 (0.78–7.65)	0.13
CCB	1.5 × 10^5^ (5.21–4.35 × 10^9^)	0.03	0.16 (0.02–1.48)	0.11
Class IC	1.2 × 10^3^ (0.00–2.91 × 10^9^)	0.34	0.46 (0.04–5.42)	0.54
Class III	0.94 (3.11 × 10^–7^–1.73 × 10^4^)	0.673	1.50 (0.23–9.83)	0.67
PVC burden (%)	0.76 (0.52–0.90)	0.16	1.02 (0.96–1.08)	0.56
Number of PVC morphologies	0.42 (0.02–7.91)	0.55	1.04 (0.65–1.67)	0.86
Unsuccessful ablation	0.01 (1.18 × 10^–6^–219.42)	0.39	1.75 (0.37–8.24)	0.48
**Ablation site**				
LV	0.01 (4.52 × 10^–6^–4.60)	0.12	3.00 (0.94–9.62)	0.06
RV	310.44 (0.34–2.83 × 10^5^)	0.09	0.42 (0.13–1.29)	0.103
Both	78.41 (1.9 × 10^–4^–2.19 × 10^7^)	0.50	0.24(0.03–3.03)	0.30
LVEDV (mL)	0.91 (0.87–0.96)	0.02	1.01 (0.99–1.03)	0.06
LVEDVi	0.69(0.62–0.75)	< 0.01	1.02 (0.99–1.04)	0.16
Scar	0.06 (9.30 × 10^–6^–369.44)	0.518	0.95(0.24–3.80)	0.95
Scar burden (%)	0.82 (0.39–1.75)	0.606	0.98 (0.87–1.10)	0.73
Global longitudinal strain (%)	3.22 (1.67–6.12)	< 0.01	0.79 (0.68–0.93)	< 0.01
Max strain rate	1.31 (0.02–102.31)	0.90	1.01 (0.97–0.35)	0.97

Of the 10 patients with scar, four patients had transmural scars consistent with an ischemic pattern; two of these patients had preserved EF and two had reduced EF. One of the two cardiomyopathy patients with ischemic scar was lost to follow-up, and one had an unsuccessful ablation but had improved function after the procedure.

From the 10 patients with scar, 9 (90%) had ablations at the scar site, and 3 (30%) had unsuccessful ablations (Table 4).

**Table 4:** Description of patients with scar noted on CMR.

	Scar description	Ischemic	PVC morphology	Ablation site	Successful ablation	EF, pre (%)	EF, post
1	Mid-myocardial at base and midportion of the RV free and anterior wall, mid-myocardial at inferior LV base	No	3	RV free wall	Yes	60	–
			LBBB	RV epicardium			
2	75% thickness of myocardium from lateral basal wall through mid-myocardium, over length of 3.6 cm	No	3	RV, posterior pap muscle	Yes	50	–
			LBBB				
			LBBB				
			RBBB				
3	Mid LV through base, inferior and inferolateral wall	Yes	1	LV, mid-inferoapical	Yes	60	–
			RBBB				
4	Small, mesocardial in inferolateral mid-ventricular wall	No	1	LV, LVOT	Yes	55	–
			LBBB				
5	Mid LV, subendocardial 50% to transmural; apex: transmural scar in anteroseptal wall	Yes	1	LV, attempted below aortic cusps	No	56	–
			RBBB				
6	Minimal, insertion site of posterior RV with RV strain	No	1	RV, septum	Yes	45	–
			LBBB				
7	Subendocardial, inferolateral wall at apex, 75% transmural	Yes	1	LV, apex	Yes	30	–
			RBBB				
8	Subepicardial 50% transmural in basilar inferoseptal wall	Yes	1	None attempted	No	40	65%
			LBBB				
9	Linear enhancement at base and mid-septum sparing the subendocardium	No	2	LV, inferior septum at scar	No	35	50%
			RBBB				
			RBBB				
10	mid LV with subendocardial 25% thickness	No	1	LV, Postero-lateral papillary muscle	Yes	45	50%
			RBBB				

#### Strain analysis

The maximum longitudinal strain was closely correlated with the ejection fraction (r = 0.615, *p* < 0.001). Average global longitudinal strain in the cardiomyopathy group was 16.19% ± 4.50%, and 9.0% ± 7.09% in the preserved EF group. Max strain rate was not significantly correlated with any measure.

Global longitudinal strain was not significantly correlated with scar burden (r = − 0.010, *p* = 0.49).

#### Volumetric measurements

Left ventricular end-diastolic volume (LVEDV) in the cardiomyopathy group was 175.43 ± 77.22 mL and 140.50 ± 36.93 mL in the preserved EF group. There was a significant correlation with ejection fraction (OR 0.613, *p* < 0.001). In investigating the relationship of LVEDV on EF recovery in the cardiomyopathy group, there was no significant correlation.

### PVC burden

The average PVC burden was 22 ± 10.4%, and this was not significantly different between the cardiomyopathy and preserved EF groups. Most patients were observed to have one PVC morphology, and average number of PVC morphologies between the two groups was not significantly different.

Similarly, in evaluation of cardiac function after PVC ablation, there was no significant correlation between either PVC burden or number of PVC morphologies and recovery of EF after ablation.

### EP study and ablation

In total, 8 (15.7%) of the patients had PVCs that were either unable to be ablated due to proximity to essential structures or native conduction pathways or that were refractory to ablation. There was not a significant difference between the two groups with regards to whether or not the PVC origin was amenable to ablation. In patients with multiple PVC morphologies, the dominant PVC morphology (determined by prior ECG, event monitor, or inpatient telemetry) was targeted. In total, 21 (41.2%) of patients had ablations in the left ventricle (LV), 23 (45.1%) had ablations in the RV, and 4 (7.8%) had ablations in both ventricles. The preserved EF group had a statistically significant higher incidence of PVC origin in RV (56.0% vs. 34.6%, *p* = 0.03). In evaluating post-procedure cardiac function of the cardiomyopathy group, PVC origin in the RV was negatively correlated recovery of EF after ablation (OR 0.050, *p* = 0.03).

Two patients had epicardial ablations, one in the LV and one in the RV, both of which were successful procedures. The patient undergoing RV epicardial ablation had preserved EF, and the patient with the LV epicardial ablation had reduced EF without recovery of function.

Of the unsuccessful ablations, four were in the LV, two were in the RV, and two had scar localized to the LV with no ablation attempted due to proximity to native conduction system. Five patients who had unsuccessful or partially successful ablations recovered function after ablation. There was no correlation between myocardial scar and unsuccessful ablations (OR 3.086, *p* = 0.179).

## Discussion

In this retrospective cohort study of patients undergoing PVC ablation with pre-procedural CMR, we found (1) patients with PVC CM had a larger LVEDV, reduced maximum longitudinal strain and were less likely to have a predominant PVC orginating from the RV, (2) there was no significant association of LV scar burden with recovery of LV function, (3) there was no significant baseline CMR functional strain parameter that predicts recovery of LVEF after PVC ablation and (4) PVC origin within the RV was associated with lack of EF recovery after PVC ablation.

PVC CM remains a diagnosis of exclusion. While patients with known scar are more prone to ventricular arrhythmia, the lack of LGE in our patient cohort as well as the lack of correlation between scar and clinical predictors suggests PVC CM is not a scar mediated process. CMR as an investigative tool in patients with purported idiopathic cardiomyopathy remains very important to rule out other reversible etiologies. Previous papers have described extracellular volume (ECV) evaluated in CMR T1 phase as correlating with both PVC-induced cardiomyopathy and with PVC burden^10,11^. This, combined with our finding of no clinical correlation with T2 LGE suggests that PVC CM is more likely a diffuse process, causing or stemming from diffuse fibrosis, rather than a focal, fibrotic, scar-forming process.

Nearly all patients with ischemic cardiomyopathy form myocardial scar, which is visualized as delayed enhancement on CMR. However, amongst patients with non-ischemic cardiomyopathy (NICM), the frequency has been demonstrated at 12%^12^. Amongst patients with PVC CM and ruled out for CAD, this incidence has been shown to be anywhere from 7 to 41%^13,14^. We found that in our cohort, 19.6% of patients had LGE present, of which 60% was in a non-ischemic pattern. However, the presence of scar, ischemic or otherwise, was evenly distributed between the cardiomyopathy and preserved EF groups. Furthermore, neither the presence of scar nor the scar burden was significantly associated with recovery of EF after ablation. PVC origin was highly co-localized with the scar site on CMR. Both of these findings are consistent with prior studies^13^. In a study assessing utility of pre-procedural CMR before PVC ablation, there was some predictive value of mortality in patients with scar and inducible VT on EP study^15^, but this study did not exclude patients with known ischemic disease, as we did in our cohort.

Global longitudinal strain has previously been shown to be a significant predictor of mortality, with estimates as high as 89.1% increase in death per each 1% of worsened strain in patients with cardiomyopathy^16^. Longitudinal strain and ejection fraction as a continuous variable are unsurprisingly significantly correlated, with our data suggesting an increase in EF of 7.4% per 4.9% increase in longitudinal strain. However, the lack of significant correlation between longitudinal strain and recovery of EF in our study is interesting. Longitudinal strain assessed by speckle tracking on TTE has been well-established to predict recovery of LV function in cases of ischemic cardiomyopathy after revascularization or medical management of ischemia^17,18^. In patients with non-ischemic cardiomyopathy hospitalized for new-onset heart failure, the utility of longitudinal strain as predictor for recovery of EF has also been demonstrated^19^.

The difference in these results compared with our findings may be attributed to the degree of cardiac decompensation. In the study by Swat et al. patients were recruited during hospitalization for heart failure symptoms, while the majority of patients in our cohort were not symptomatic from heart failure, instead citing syncope or palpitations as reason for pursuing ablation. It is notable that the average longitudinal strain in this study was 15.2% in persistently reduced EF patients and 16.5% in recovered EF patients, while the average longitudinal strain of normal EF patients was 20.6%. It may be that the relation of longitudinal strain to EF recovery is more strongly correlated in decompensated heart failure.

In addition, the development of PVC-induced cardiomyopathy is thought related to how long a patient has had PVCs. Several studies have shown asymptomatic presentation as a risk for development of PVC CM^4,20,21^, thought likely because these patients do not seek care and have prolonged duration of exposure to PVCs. In the study by Swat et al. the mean time to EF recovery was 135 days. The majority of the patients in our cohort have had PVCs for years, most of whom have also failed medical management. Thus, it may also be that longitudinal strain is less predictive with chronic changes. Finally the use of TTE in that study compared to the use of CMR in our study to assess longitudinal strain should be noted but is unlikely to explain the discrepancy.

Several studies have attempted to quantify a threshold of PVC burden at which patients were likely to develop PVC CM. Baman et al. introduced a threshold of PVC > 24%, with a sensitivity of 79% and specificity of 78%^3^. This threshold was later challenged, with a study showing a threshold of PVC burden > 16% (sensitivity 100%, specificity 87%)^4^. In a canine model, PVC CM occurred at a PVC burden as low as 11% in 25% of subjects, but 100% of subjects had impaired LV function at a PVC burden of 50%^5^. Though thresholds have been defined, there have been outliers and PVC CM has been reported with a burden as low as 4%^22^.

In our study we did not find PVC burden to be a statistically significant predictor of either reduced EF or subsequent recovery of function. However, in our cohort of patients the overall PVC burden was high, thus limiting comparisons. Both the lack of correlation between PVC burden with the presence of PVC CM as well as with recovery of EF have been previously documented^23,24^. Furthermore, studies have shown correlation of the temporal heterogeneity of PVCs^25^ or burden of interpolated PVCs to development of cardiomyopathy^1,7,20^ suggesting that there may be more complexity to the issue than pure PVC burden.

In our study, we observed a higher incidence of PVC origins in the RV amongst patients with preserved EF. It was previously shown that PVCs arising from the RV, predominantly the RV outflow tract (RVOT) are more likely to cause cardiomyopathy^23^. However, subsequent studies have demonstrated that there is not a statistically significant difference in whether PVCs arise from the LV or RV^3,26,27^. In the cardiomyopathy group, there was an observed statistically significant correlation of RV origin with persistently reduced EF. RV ablations, specifically RVOT ablations, have been shown to have the highest success rate^6^. This was supported by our findings as well, with 6 of the 8 unsuccessful ablations localizing PVC origin to the LV. However, these patients were split equally between the cardiomyopathy and preserved EF groups.

With regards to the relationship between PVC origin site and EF recovery, a prior study showed a higher incidence of EF recovery of PVC CM in LV ablations compared to RV ablations, though this was not a statistically significant finding^28^. Our study corroborates this with a significant correlation between RV site and lack of EF recovery. It may be that with an RV site of origin leads to significant ventricular dyssynchrony, similar to what is observed with left bundle branch bock, and thus patients are at higher risk for developing irreversible cardiomyopathy.

In this study, we did not exclude unsuccessful or partially successful ablations. Interestingly, of the 8 ablations that were not successful, 3 did not have reduced ejection fractions and 5 recovered function despite the lack of procedural success. As PVC cardiomyopathy is technically defined as cases with improvement of EF after resolution of PVCs, these cases do not qualify for the diagnosis.

### Study limitations

There were several limitations in this study. First, our medical center is a quaternary care center and receives referrals from all over the world. Thus, there was limitation in access to medical records both pre-ablation, as well as post-ablation follow-up. A major outcome, post-ablation improvement in EF, was not able to be assessed in all cardiomyopathy patients due to lack of records. As previously mentioned, description of ECV correlating with PVC burden and PVC CM has been previously reported. We were unable to include this description in our study as ECV assessment is not part of the clinical protocol at our institution. The sample size of this study was limited due in part to the nature of PVC cardiomyopathy being a diagnosis of exclusion and a minority of patients are referred for catheter ablation. Another limitation was with our CMR image standardization, as patients oftentimes completed CMR imaging at outside facilities. This was addressed as best as possible by excluding incomplete image series.

## Conclusion

In this cohort of patients undergoing CMR prior to a PVC ablation, and without history of ischemic or structural heart disease, there were no significant CMR findings to predict the presence of PVC CM or to predict subsequent recovery of EF after ablation. The burden of existing scar is was not associated with either endpoint, nor was the magnitude of global longitudinal strain. PVC origin in the RV was associated with lack of EF recovery after ablation and further investigation into the mechanism of this process is warranted.
